# Nucleus Accumbens Dopamine D1-Receptor-Expressing Neurons Control the Acquisition of Sign-Tracking to Conditioned Cues in Mice

**DOI:** 10.3389/fnins.2018.00418

**Published:** 2018-06-21

**Authors:** Tom Macpherson, Takatoshi Hikida

**Affiliations:** ^1^Laboratory for Advanced Brain Functions, Institute for Protein Research, Osaka University, Osaka, Japan; ^2^Medical Innovation Center, Graduate School of Medicine, Kyoto University, Kyoto, Japan

**Keywords:** dopamine receptor, autoshaping, striatum, reward learning, reversible neurotransmission blocking

## Abstract

Following repeated pairings, the reinforcing and motivational properties (incentive salience) of a reward can be transferred onto an environmental stimulus which can then elicit conditioned responses, including Pavlovian approach behavior to the stimulus (a sign-tracking response). In rodents, acquisition of sign-tracking in autoshaping paradigms is sensitive to lesions and dopamine D1 receptor antagonism of the nucleus accumbens (NAc) of the ventral striatum. However, currently, the possible roles of dorsal striatal subregions, as well as of the two major striatal neuron types, dopamine D1-/D2-expressing medium spiny neurons (MSNs), in controlling the development of conditioned responses is still unclear and warrants further study. Here, for the first time, we used a transgenic mouse line combined with striatal subregion-specific AAV virus injections to separately express tetanus toxin in D1-/D2- MSNs in the NAc, dorsomedial striatum, and dorsolateral striatum, to permanently block neurotransmission in these neurons during acquisition of an autoshaping task. Neurotransmission blocking of NAc D1-MSNs inhibited the acquisition of sign-tracking responses when the initial conditioned response for each conditioned stimulus presentation was examined, confirming our initial hypothesis. These findings suggest that activity in NAc D1-MSNs contributes to the attribution of incentive salience to conditioned stimuli.

## Introduction

Transference of the motivational and reinforcing properties (incentive salience) of a rewarding unconditioned stimulus (US) onto a previously neutral conditioned stimulus (CS), following repeated pairings, can result in the CS eliciting conditioned responses, including approach behavior, in some individuals (Robinson and Berridge, 1993, 2001). In an “autoshaping” paradigm, presentation of a CS associated with a reward (CS+), but not a CS associated with no consequence (CS-), can result in “sign-tracking” approach behavior, and has been used to model appetitive Pavlovian conditioning in rodents (Bussey et al., 1997; Cardinal et al., 2002). Dopamine release in the nucleus accumbens (NAc) is critical for learning CS–US associations that result in sign-tracking (Dalley et al., 2002; Parkinson et al., 2002; Flagel et al., 2010), and while cue-evoked dopamine release in the NAc is reduced following extended training, Pavlovian approach behavior is still dependent on NAc dopamine (Fraser and Janak, 2017). Within the striatum, dopamine D1 and D2 receptors are expressed on largely separate medium spiny neuron (MSN) populations, and neurotransmission in NAc D1- and D2-MSNs is necessary for reward or aversion learning, respectively, in drug- or food- conditioned place preference (CPP) and shock-induced conditioned place aversion (CPA) tasks (Gerfen and Young, 1988; Gerfen et al., 1990; Hikida et al., 2010; Lobo et al., 2010; Macpherson et al., 2014).

In the dorsal striatum, subregions can be functionally delineated, and lesions of the dorsomedial striatum (DMS), but not the dorsolateral striatum (DLS), impair acquisition of a sucrose CPP task, while inhibition of neural activity or dopamine receptor antagonism in the DLS does not alter the expression of sign-tracking in autoshaping (Everitt et al., 1991; Fraser and Janak, 2017). Interestingly, optogenetic activation of D1- and D2-MSNs in the dorsal striatum is sufficient to induce CPP or CPA, respectively (Kravitz et al., 2012).

Another type of conditioned response, known as “goal-tracking,” has also been reported in rodents and describes approach behavior toward the location of the US delivery, despite the US not being delivered until after the termination of the CS (Boakes, 1977). While NAc dopamine antagonism has no effect on the expression of goal-tracking, the role of the dorsal striatum in controlling sign-tracking is less clear (Saunders and Robinson, 2012).

Recently, systemic D1 antagonism was shown to inhibit the acquisition of sign-tracking, but facilitate goal-tracking, while systemic D2 antagonism produced no effect on either response (Chow et al., 2016). Similarly, intra-NAc D1, but not D2, antagonism inhibited the acquisition of sign-tracking (Dalley et al., 2005). Here, we investigated reversible neurotransmission blocking (RNB) of D1-/D2-MSNs in the NAc, DMS, or DLS on the initial conditioned response to each CS+/CS- presentation during the acquisition of an autoshaping task, and hypothesized that NAc D1-RNB mice will similarly show impaired acquisition of sign-tracking and facilitated goal-tracking.

## Methods

### Animals

Male D1-/D2-MSN-inhibited (D1-/D2-RNB) and wild type (WT) mice aged between 10 and 12 weeks were generated using tetenus neurotoxin (TeNT) transgenic mice on a C57BL/6 background. TeNT is a bacterial toxin that cleaves the synaptic-vesicle-associated VAMP2 protein and abolishes neurotransmitter release from the synaptic vesicles of the target neurons (Schiavo et al., 1992; Wada et al., 2007). In TeNT mice, the expression of TeNT is under the control of tetracycline-responsive element (TRE) and is driven by the interaction of TRE with tetracycline-repressive transcription factor (tTA) in the absence of doxycycline (Hikida et al., 2010). tTA expression was restricted to either D1-MSNs [coexpressing Substance P (SP)] or D2-MSNs [coexpressing Enkephalin (Enk)] by intracranial infusion of two recombinant AAV viruses (AAV2-SP-tTA or AAV2-Enk-tTA, described in Hikida et al., 2010). Following anesthesia (90 mg/kg Ketamine and 20 mg/kg Xylazine, i.p. injection), AAV2-SP-tTA or AAV2-Enk-tTA was bilaterally delivered into two sites (500 nl/site at 100 nl/min; left for 5 min) in the NAc (WT *n* = 11, D1-RNB *n* = 8, D2-RNB *n* = 8), DMS (WT *n* = 15, D1-RNB *n* = 12, D2-RNB *n* = 11), or DLS (WT *n* = 14, D1-RNB *n* = 9, D2-RNB *n* = 9) of WT, D1-, and D2-RNB mice. Stereotaxic coordinates were: NAc, AP +1.4, L ± 0.8 and DV -4.0 and -3.5; DMS, AP +0.3 and +0.9, L ± 1.5 and DV -2.5; DMS, AP +0.3 and +0.9, L ± 2.5 and DV -3.0 (spread of ±0.5 mm in each area), based on those used in Hikida et al. (2010) for the NAc, and (Gremel and Costa, 2013) for the DMS and DLS, and were histologically verified following completion of the experiment (see **Supplementary Figure S1**). One mouse was excluded from the analysis due to a misaligned injection site. We previously confirmed that WT mice show no behavioral changes after infusion of these viruses, and therefore received either AAV2-SP-tTA or AAV2-Enk-tTA in approximately equal numbers and were grouped for analysis as a control group (Hikida et al., 2010).

After surgery, mice underwent a 2-week recovery/expression period during which they were observed daily for signs of discomfort/disability before the commencement of behavioral procedures. From birth, and extending throughout the duration of the experiment, all mice remained off doxycycline, resulting in continuous neurotransmission blocking in D1- and D2-RNB mice. Mice were housed in groups of 2–3 in cages containing woodchip bedding and cardboard nesting material, and were maintained on a 12-h light/dark cycle (lights on at 8:00 a.m.) and the temperature controlled to 24 ± 2°C in a humidity of 50 ± 5%. All animal handling procedures and use of viral and tetanus toxin constructs were approved by the animal research committees of Kyoto University Graduate School of Medicine and Institute for Protein Research, Osaka University.

### Autoshaping

Beginning 3 days prior to, and continuing throughout the experiment, water drinking was restricted to a 2-h session per day (17:30–19:30) to motivate the mice to seek a liquid reward. During this time, mice were observed daily for signs of dehydration. Mice were trained in trapezoidal touch-screen operant chambers (Campden Instruments, Ltd., United Kingdom) housed within a light-resistant, sound-attenuating cubicle. Chambers were equipped with a liquid reward magazine located in the middle of a front touchscreen panel. Mice underwent two 40 min magazine training/habituation sessions on consecutive days, during which they learnt to collect a 2 ml 10% condensed milk solution delivered at the beginning of the session. Next, animals underwent six consecutive daily conditioning sessions, during which they were presented with a 10 s visual stimuli (white panel) on either the left or right side of the front panel. One side was designated the CS+ and was paired with 500 μl 10% condensed milk delivered immediately after the termination of the illuminated panel, while the other side was designated the CS- and was associated with no outcome. Assignment of CS+/CS- side was counterbalanced among animals. Each CS+/CS- presentation was followed by a 10–40 s variable inter-trial-interval (ITI), and mice were required to break a photocell beam spanning the entirety of the back of the chamber to initiate the next trial, ensuring they were able to attend to both sides of the screen. Each daily session consisted of 40 trials composed of 20 presentations of each the CS+ and CS- in a randomized order, with never more than two consecutive presentations of each CS. Based upon the protocol presented in Horner et al. (2013), we analyzed the initial conditioned response during each CS trial, and thus a sign-tracking response was recorded when the mouse broke a photocell beam located 3 cm in front of the CS+/CS- before any entry into the reward magazine, and a goal-tracking response when the mouse broke a photocell beam located within the reward magazine before any CS+/CS- approach. Responses subsequent to the initial response were not recorded.

### Data Analysis

**FIGURE 1:**
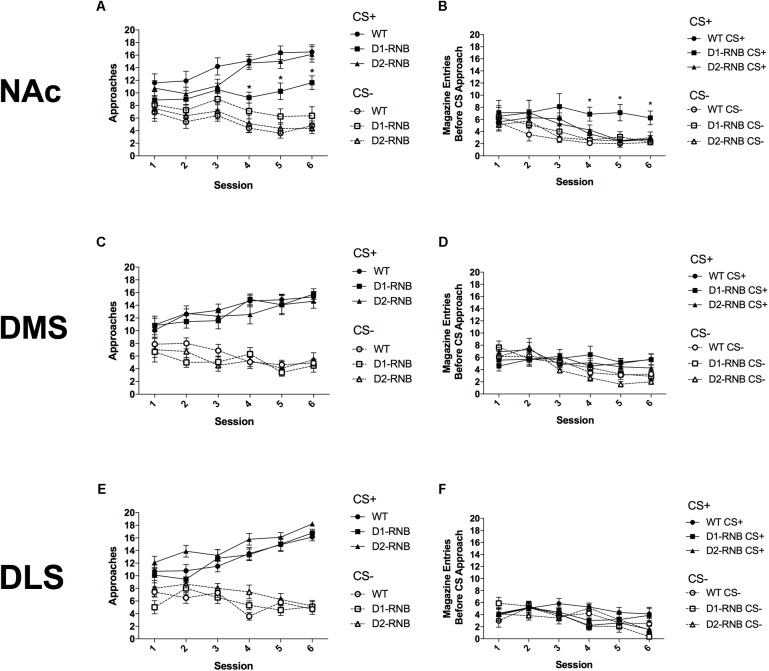
Autoshaping in striatal RNB mice. **(A)** Sign-tracking in NAc RNB mice. NAc D1-RNB showed reduced sign-tracking. **(B)** Goal-tracking in NAc RNB mice. NAc D1-RNB mice showed increased goal-tracking. NAc, nucleus accumbens. Sign-tracking in **(C)** DMS and **(E)** DLS RNB mice. Goal-tracking in **(D)** DMS and **(F)** DLS RNB mice. DMS, dorsomedial striatum; DLS, dorsolateral striatum. Values represent mean ± SEM, ^∗^*p* < 0.05 compared with WT and D2-MSN mice in Bonferroni *post hoc* comparisons.

Data are expressed as mean ± SEM. Session totals of the initial conditioned response (sign-tracking or goal-tracking) for each of 20 CS+ and 20 CS- trials were analyzed using three-way repeated measures ANOVAs (see **Table 1**), and two-way ANOVAs and Bonferroni *Post hoc* comparisons for each individual session (see **Figure 1**).

**Table 1:** Statistical analyses of sign-tracking and goal-tracking in NAc, DMS, and DLS RNB mice.

Reference	Variable	*F*	Significance
**Figure 1A**	CS	*F*_(1,115)_ = 93.68	*p* < 0.001^∗∗^
**Figure 1A**	Session	*F*_(5,115)_ = 2.57	*p* = 0.030^∗^
**Figure 1A**	Genotype	*F*_(2,23)_ = 0.80	*p* = 0.460
**Figure 1A**	CS^∗^Session	*F*_(5,115)_ = 14.53	*p* < 0.001^∗∗^
**Figure 1A**	CS^∗^Genotype	*F*_(2,115)_ = 9.39	*p* < 0.001^∗∗^
**Figure 1A**	Session^∗^Genotype	*F*_(10,115)_ = 0.45	*p* = 0.919
**Figure 1A**	CS^∗^Session^∗^Genotype	*F*_(10,115)_ = 1.30	*p* = 0.237
**Figure 1B**	CS	*F*_(1,115)_ = 13.45	*p* < 0.001^∗∗^
**Figure 1B**	Session	*F*_(5,115)_ = 9.25	*p* < 0.001^∗∗^
**Figure 1B**	Genotype	*F*_(2,23)_ = 8.57	*p* = 0.002^∗^
**Figure 1B**	CS^∗^Session	*F*_(5,115)_ = 0.44	*p* = 0.819
**Figure 1B**	CS^∗^Genotype	*F*_(2,115)_ = 6.54	*p* < 0.001^∗∗^
**Figure 1B**	Session^∗^Genotype	*F*_(10,115)_ = 0.52	*p* = 0.875
**Figure 1B**	CS^∗^Session^∗^Genotype	*F*_(10,115)_ = 0.51	*p* = 0.881
**Figure 1C**	CS	*F*_(1,175)_ = 290.73	*p* < 0.001^∗∗^
**Figure 1C**	Session	*F*_(5,175)_ = 3.38	*p* = 0.006^∗^
**Figure 1C**	Genotype	*F*_(2,35)_ = 0.80	*p* = 0.460
**Figure 1C**	CS^∗^Session	*F*_(5,175)_ = 21.68	*p* < 0.001^∗∗^
**Figure 1C**	CS^∗^Genotype	*F*_(2,175)_ = 0.30	*p* = 0.743
**Figure 1C**	Session^∗^Genotype	*F*_(10,175)_ = 1.33	*p* = 0.219
**Figure 1C**	CS^∗^Session^∗^Genotype	*F*_(10,175)_ = 2.41	*p* = 0.992
**Figure 1D**	CS	*F*_(1,175)_ = 21.53	*p* < 0.001^∗∗^
**Figure 1D**	Session	*F*_(5,175)_ = 7.64	*p* < 0.001^∗∗^
**Figure 1D**	Genotype	*F*_(2,35)_ = 0.02	*p* = 0.985
**Figure 1D**	CS^∗^Session	*F*_(5,175)_ = 8.62	*p* < 0.001^∗∗^
**Figure 1D**	CS^∗^Genotype	*F*_(2,175)_ = 2.34	*p* = 0.112
**Figure 1D**	Session^∗^Genotype	*F*_(10,175)_ = 1.16	*p* = 0.320
**Figure 1D**	CS^∗^Session^∗^Genotype	*F*_(10,175)_ = 0.04	*p* = 0.847
**Figure 1E**	CS	*F*_(1,145)_ = 374.07	*p* < 0.001^∗∗^
**Figure 1E**	Session	*F*_(5,145)_ = 4.03	*p* = 0.002^∗^
**Figure 1E**	Genotype	*F*_(2,29)_ = 1.23	*p* = 0.307
**Figure 1E**	CS^∗^Session	*F*_(5,145)_ = 12.48	*p* < 0.001^∗∗^
**Figure 1E**	CS^∗^Genotype	*F*_(2,145)_ = 0.23	*p* = 0.799
**Figure 1E**	Session^∗^Genotype	*F*_(10,145)_ = 0.97	*p* = 0.473
**Figure 1E**	CS^∗^Session^∗^Genotype	*F*_(10,145)_ = 1.26	*p* = 0.261
**Figure 1F**	CS	*F*_(5,145)_ = 9.36	*p* < 0.001^∗∗^
**Figure 1F**	Session	*F*_(5,145)_ = 6.52	*p* < 0.001^∗∗^
**Figure 1F**	Genotype	*F*_(2,29)_ = 0.66	*p* = 0.525
**Figure 1F**	CS^∗^Session	*F*_(5,145)_ = 1.17	*p* = 0.210
**Figure 1F**	CS^∗^Genotype	*F*_(2,145)_ = 2.14	*p* = 0.138
**Figure 1F**	Session^∗^Genotype	*F*_(10,145)_ = 0.92	*p* = 0.511
**Figure 1F**	CS^∗^Session^∗^Genotype	*F*_(10,145)_ = 0.08	*p* = 0.832

*^∗^p < 0.05, ^∗∗^p < 0.001.*

## Results

### The Role of NAc MSNs in Controlling Autoshaping

Over six conditioning sessions, CS+ approaches increased and CS- approaches decreased in all mice (**Figure 1A** and **Table 1**; Significant CS^∗^Session interaction (*F*_(5,115)_ = 14.53, *p* < 0.001). However, by the final three conditioning sessions, CS+ approaches were reduced in NAc D1-RNB mice compared with both WT and NAc D2-RNB mice (**Figure 1A**; Bonferroni *post hoc* analyses of each session). Interestingly, CS+ approaches by NAc D1-RNB mice were still significantly greater than CS- approaches during the final two conditioning sessions (**Figure 1A**; Two-way repeated measures ANOVA split by genotype, D1-RNB; Significant main effect of CS on Day 5 (*F*_(1,7)_ = 5.97, *p* = 0.044) and Day 6 (*F*_(1,7)_ = 6.03, *p* = 0.044).

Conversely, both CS+ and CS- goal-tracking responses decreased over six sessions (**Figure 1B** and **Table 1**; Significant main effect of Session (*F*_(5,115)_ = 9.25, *p* < 0.001), Non-significant CS^∗^Session interaction (*F*_(5,115)_ = 0.44, *p* = 0.819). However, by the final three sessions CS+ responses in NAc D1-RNB mice were greater than those in WT and D2-RNB counterparts (**Figure 1B**; Bonferroni *post hoc* analyses of each session), and were greater than CS- responses (**Figure 1B**; Two-way repeated measures ANOVA split by genotype, D1-RNB; Significant main effect of CS on Day 4 (*F*_(1,7)_ = 6.68, *p* = 0.036), Day 5 (*F*_(1,7)_ = 10.18, *p* = 0.015), and Day 6 (*F*_(1,7)_ = 6.89, *p* = 0.034).

These data indicate that NAc D1-MSN neurotransmission contributes to the acquisition of sign-tracking and that inhibition of NAc D1-MSNs facilitates goal-tracking responses, while NAc D2-MSN neurotransmission is not necessary for sign-tracking or goal-tracking.

### The Role of Dorsal Striatal MSNs in Controlling Autoshaping

Inhibition of D1- or D2-MSNs in either the DMS or DLS did not alter sign-tracking acquisition, as indicated by increased CS+ approaches and decreased CS- approaches, regardless of genotype, over six sessions (**Figures 1C,E** and **Table 1**; Significant CS^∗^Session interaction [DMS; (*F*_(5,175)_ = 21.68, *p* < 0.001), DLS; (*F*_(5,145)_ = 12.48, *p* < 0.001)], Non-significant CS^∗^Session^∗^Genotype interaction [DMS; (*F*_(10,175)_ = 2.41, *p* = 0.992), DLS; (*F*_(10,145)_ = 1.26, *p* = 0.261)].

In DMS RNB mice, CS-, but not CS+ goal-tracking responses decreased over six sessions and similarly did not differ among genotypes (**Figure 1D** and **Table 1**; DMS, Significant main effect of Session (*F*_(5,175)_ = 7.64), *p* < 0.001), Significant CS^∗^Session interaction (*F*_(5,175)_ = 8.62, *p* < 0.001), Non-significant CS^∗^Session^∗^Genotype interaction (*F*_(10,175)_ = 0.04, *p* = 0.847). While, in DLS RNB mice, both CS+ and CS- goal-tracking responses decreased in all genotypes (**Figure 1F** and **Table 1**); Significant main effect of Session (*F*_(5,145)_ = 6.52, *p* < 0.001), Non-significant CS^∗^Session interaction (*F*_(5,145)_ = 1.17, *p* = 0.210), Non-significant CS^∗^Session^∗^Genotype interaction (*F*_(10,145)_ = 0.08, *p* = 0.832).

These results indicate that neurotransmission within dorsal striatal neurons is not necessary for sign-tracking or goal-tracking acquisition.

## Discussion

Here, for the first time, we revealed that total blockade of neurotransmission in NAc D1-MSNs was sufficient to reduce Pavlovian approaches to the CS+ when the initial response to each CS presentation was measured. This finding supports previous evidence indicating a role for NAc D1-MSNs in controlling Pavlovian conditioning in cocaine- or food-CPP (Hikida et al., 2010; Lobo et al., 2010), as well as evidence that D1 receptor antagonism inhibits sign-tracking but facilitates goal-tracking (Dalley et al., 2005; Chow et al., 2016). Interestingly, approaches to the CS+ in NAc D1-RNB were still greater than those to the CS-, similar to previous evidence that while sign-tracking responses to the CS+ were reduced by NAc dopamine-depletion (Parkinson et al., 2002) or NAc-lesions (Chang et al., 2012; Chang and Holland, 2013) in rats, they were still significantly greater than responses to the CS-. We have previously demonstrated that NAc D1-RNB mice show a normal preference for chocolate food rewards over standard lab chow and normal locomotor activity (Supplementary data in Hikida et al., 2010). Thus, rather than a general attenuation of associative learning, a decrease in the appetitive properties of the liquid reward, or an inability to move toward the cue, blockade of neurotransmission in NAc D1-MSNs appears to reduce the transference of incentive salience from the liquid reward to the CS+. Interestingly, goal-tracking following the CS+, but not the CS-, was increased in NAc D1-RNB mice. This may be resultant from an increased ability to focus on goal-tracking due to reduced distraction from the incentive salience of the CS+.

In contrast to the NAc, RNB of D1- or D2-MSNs of the DMS and DLS did not alter conditioned responses in the autoshaping task. These findings support those of a previous study demonstrating that restoration of dopamine release specifically in the dorsal striatum of dopamine-depleted mice is not sufficient for acquisition of sign-tracking (Darvas et al., 2014), and indicate that dorsal striatal neurons are not necessary for the acquisition of conditioned responses in this task.

## Author Contributions

TM and TH designed the study, analyzed and interpreted the results, and wrote the manuscript. TM performed the experiments.

## Conflict of Interest Statement

The authors declare that the research was conducted in the absence of any commercial or financial relationships that could be construed as a potential conflict of interest.

## Supplementary Material

FIGURE S1 Histological map of nucleus accumbens and dorsal striatum AAV virus infusion sites. WT, D1-, and D2-RNB mice infusion sites are all presented together.
