# Supplementary figures and images for: Nucleus Accumbens Dopamine D1-Receptor-Expressing Neurons Control the Acquisition of Sign-Tracking to Conditioned Cues in Mice
Source: Front Neurosci. 2018 Jun 21;12:418. doi: 10.3389/fnins.2018.00418 (PMC6021689; doi:10.3389/fnins.2018.00418)

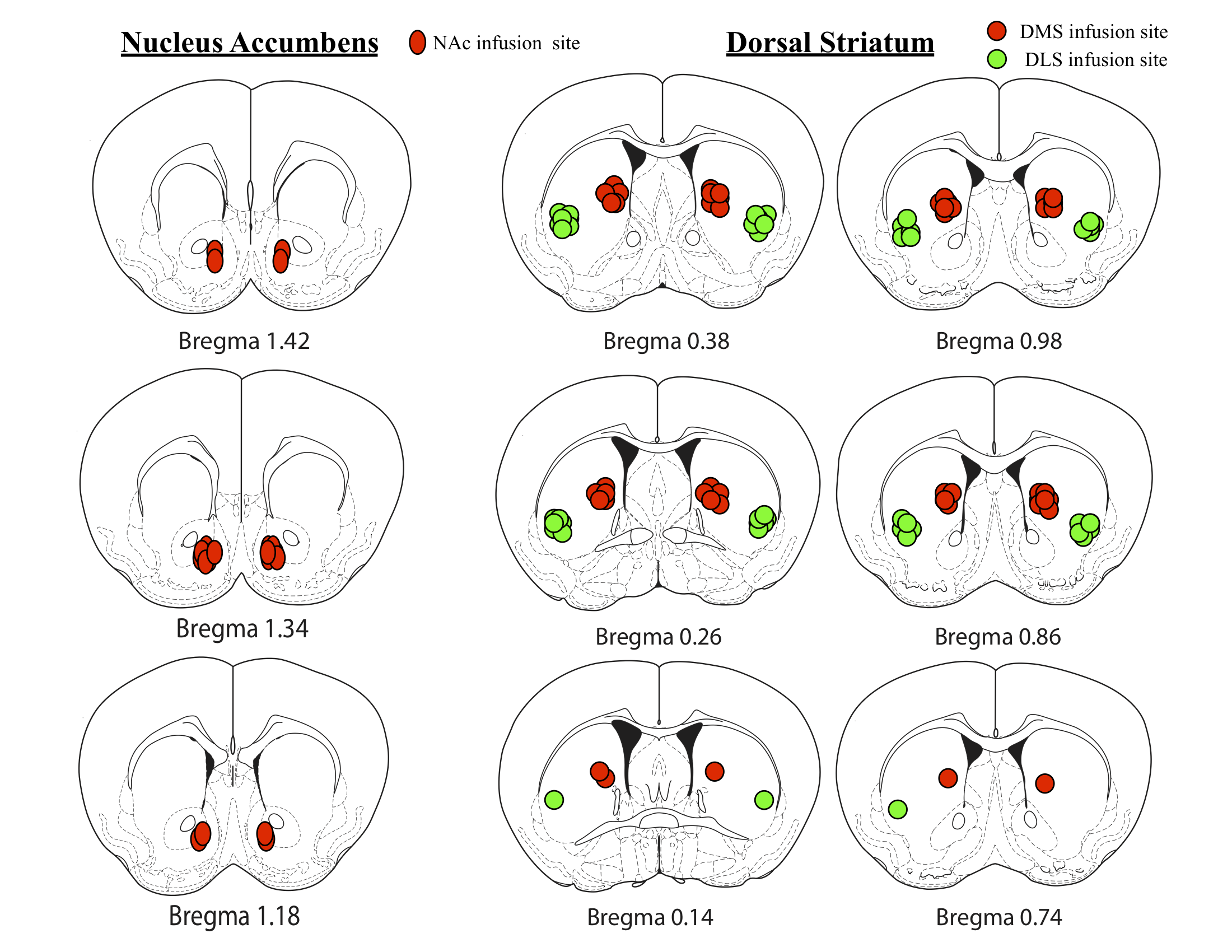

Supplement: FIGURE S1 — Histological map of nucleus accumbens and dorsal striatum AAV virus infusion sites. WT, D1-, and D2-RNB mice infusion sites are all presented together. [file Image_1.TIFF]
